# Impact of explanted valve type on aortic valve reoperations: nationwide UK experience

**DOI:** 10.1093/ejcts/ezae031

**Published:** 2024-02-01

**Authors:** Pradeep Narayan, Tim Dong, Arnaldo Dimagli, Daniel P Fudulu, Jeremy Chan, Shubhra Sinha, Gianni D Angelini

**Affiliations:** Department of Cardiac Surgery, Rabindranath Tagore International Institute of Cardiac Sciences, Narayana Health, Kolkata, India; Department of Cardiac Surgery, Bristol Heart Institute, Bristol University, Bristol, UK; Department of Cardiac Surgery, Bristol Heart Institute, Bristol University, Bristol, UK; Department of Cardiac Surgery, Bristol Heart Institute, Bristol University, Bristol, UK; Department of Cardiac Surgery, Bristol Heart Institute, Bristol University, Bristol, UK; Department of Cardiac Surgery, Bristol Heart Institute, Bristol University, Bristol, UK; Department of Cardiac Surgery, Bristol Heart Institute, Bristol University, Bristol, UK

**Keywords:** Aortic valve surgery, Reoperation, Mechanical valve, Bioprosthetic valve, In-hospital mortality, UK

## Abstract

**OBJECTIVES:**

This nationwide retrospective cohort study assessed the impact of the explanted valve type on reoperative outcomes in aortic valve surgery within the UK over a 23-year period.

**METHODS:**

Data were sourced from the National Institute for Cardiovascular Outcomes Research (NICOR) database. All patients undergoing first-time isolated reoperative aortic valve replacement between 1996 and 2019 in the UK were included. Concomitant procedures, homograft implantation or aortic root enlargement were excluded. Propensity score matching was utilized to compare outcomes and risk factors for in-hospital mortality was evaluated through multivariable logistic regression. Final model selection was conducted using Akaike Information Criterion through bootstrapping. The primary end point was in-hospital mortality, and secondary end points included postoperative morbidities.

**RESULTS:**

Out of 2371 patients, 24.9% had mechanical and 75% had bioprosthetic valves implanted during the primary procedure. Propensity matched groups of 324 patients each, were compared. In-hospital mortality for mechanical and bioprosthetic valve explants was 7.1% and 5.9%, respectively (*P* = 0.632). On multivariable logistic regression analysis, valve type was not a risk factor for mortality [odds ratio (OR) 0.62, 95% confidence interval (CI) 0.37–1.05; *P* = 0.1]. Age (OR 1.03, 95% CI 1.01–1.05; *P* < 0.05), left ventricular ejection fraction (OR 1.62, 95% CI 1.08–2.42; *P* < 0.05), creatinine ≥ 200 mg/dl (OR 2.21, 95% CI 1.17–4.04; *P* < 0.05) and endocarditis (OR 2.66, 95% CI 1.71–4.14; *P* < 0.05) emerged as risk factors for mortality.

**CONCLUSIONS:**

The type of valve initially implanted (mechanical or bioprosthetic) did not determine mortality. Instead, age, left ventricular ejection fraction, renal impairment and endocarditis were significant risk factors for in-hospital mortality.

**Figure:**
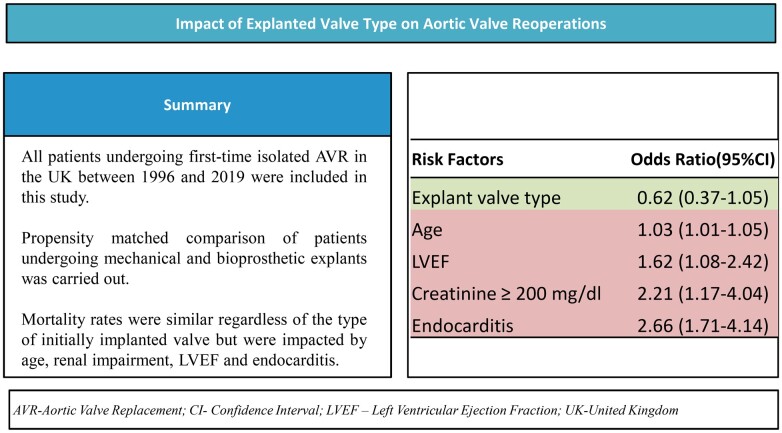

## INTRODUCTION

Reoperations on prosthetic aortic valves may be necessary due to several reasons. Structural valve degeneration primarily compels reoperation in bioprosthetic valves, whereas non-structural valve dysfunctions play a more important role for mechanical valves. The distinction between the 2 implanted valve types is further evident in their clinical presentation. Bioprosthetic valves tend to manifest a gradual and insidious deterioration in performance over time, whereas mechanical valves can give rise to more acute and emergent scenarios, often stemming from leaflet/disc malfunction or obstruction. Endocarditis, leading to the need for reoperations, can impact both types of valves and can manifest with varying degrees of urgency, regardless of the valve type used.

Despite the clinical significance of these reoperations, there is a conspicuous scarcity of comprehensive data regarding the influence of the explanted valve prosthesis on reoperative outcomes. The existing studies addressing this topic are hindered by several limitations. Many of them are confined to single-centre observations with relatively small patient cohorts [1–5]. Frequently, when analysing outcomes following explanted mechanical and biological valves, there is a lack of discrimination based on the specific location of valve implantation. As a result, studies often include aortic, mitral and tricuspid valve replacements in the comparison between mechanical and bioprosthetic valves [1, 3, 5–7]. In addition, the inclusion of second and third reoperative surgeries within these comparisons [8], as well as the concurrent performance of concomitant procedures like coronary artery bypass grafting [1, 3, 8], and aortic surgery [3], further complicates the interpretation of the findings.

This discernible gap in the literature underscores the need for a meticulous investigation into the outcomes of reoperative isolated aortic valve replacement (AVR), with specific attention to the previously implanted valve type. To address this knowledge void, the aim of this study was to assess the impact of implanted valve type on outcomes. We also sought to identify the predictors of adverse outcomes in this cohort of patients undergoing following first-time, reoperative, isolated AVR.

## PATIENTS AND METHODS

### Ethics statement

The current investigation is an integral component of a research initiative endorsed by the Health Research Authority and Health and Care Research Wales (HCRW). IRB approval was obtained and the need for individual patient consent was waived off (HCRW) (IRAS ID: 257758) amendment 2, on April 01, 2022.

Data utilized in this study were meticulously sourced from the National Institute for Cardiovascular Outcomes Research (NICOR) central cardiac database, which systematically accumulates prospective data as an integral part of the National Adult Cardiac Surgery Audit Database. The NICOR registry, serving as a comprehensive repository, captures a spectrum of demographic, intraoperative, postoperative mortality and morbidity data pertaining to all adult cardiac surgical procedures conducted across the UK. A comprehensive description of the procedural workflow, spanning from data input to analytical stages, has been previously elucidated [9]. The rate of missing data concerning baseline information within the NICOR registry remained exceedingly low, at 1.7%. For variables categorized as categorical or dichotomous, missing data points underwent imputation using the mode, while continuous variable data characterized by gaps were imputed with the median value.

**Figure 1:**
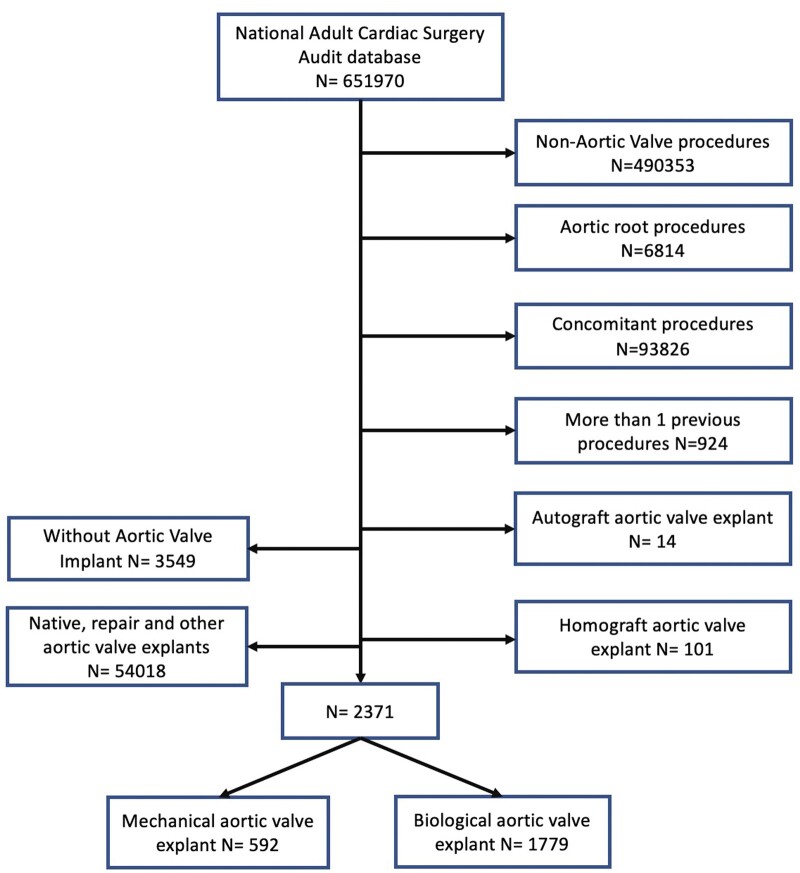
CONSORT (Consolidated Standards of Reporting Trials) diagram of the study.

All patients undergoing first-time, isolated, reoperative AVR between January 1996 and March 2019 in the UK were included in the study. Patients undergoing concomitant procedures, homograft implantation, aortic root enlargement during reoperative surgery and those where more than one previous operation was carried out were excluded from the study. A CONSORT (Consolidated Standards of Reporting Trials) diagram of the study has been provided in Fig. 1.

### Outcomes

The primary end point under investigation was in-hospital mortality. Secondary end points included neurological complications encompassing both transient ischaemic attacks and strokes, the requirement for postoperative dialysis, return to operating theatre for bleeding, incidence of deep sternal wound infections, postoperative intra-aortic balloon pump usage and the duration of postoperative stay.

### Statistical methods

We utilized normal quantile plots to evaluate the adequacy of a normal distribution model for continuous variables. For continuous variables, we assessed centrality and dispersion using the median and interquartile range. Categorical variables were summarized by presenting frequency and percentage (%). To assess disparities in baseline characteristics between the 2 groups, we applied Kruskal–Wallis rank sum tests for continuous variables and Pearson’s χ^2^ test for categorical variables. For exploratory analysis of trend, the association between in-hospital mortality and year of operation was modelled by natural cubic splines (Supplementary Material, Fig. S1).

The distribution of propensity scores was compared against that of inverse probability of treatment weighting in the Supplementary Material, Fig. S2. While inverse probability of treatment weighting may be useful in reducing data attrition, it can be seen that inverse probability of treatment weighting is sensitive to extreme propensity values and was not selected in this particular study [10]. A propensity score was calculated using a comprehensive logistic regression model that included various covariates: age, gender, body mass index, New York Heart Association (NYHA) class, Canadian Cardiovascular Society grading, diabetes mellitus, hypertension, smoking history, prior myocardial infarction, chronic kidney disease (taken as creatinine >200 mg/dl), chronic obstructive pulmonary disease, prior cerebrovascular accident, peripheral vascular disease, preoperative atrial fibrillation, left ventricular ejection fraction (LVEF), aortic valve haemodynamic, aortic valve endocarditis, aortic valve implant type and the year of the operation.

The binary dependent variable in this analysis represented the treatment effect, distinguishing reoperative mechanical valve replacement from reoperative biological valve replacement. Propensity score matching was then employed to evaluate this treatment effect. Pairs of patients were meticulously matched in a 1:1 ratio, with a caliper width set at 0.05 standard deviations of the logit of the propensity score, utilizing the nearest-neighbour method.

**Figure 2:**
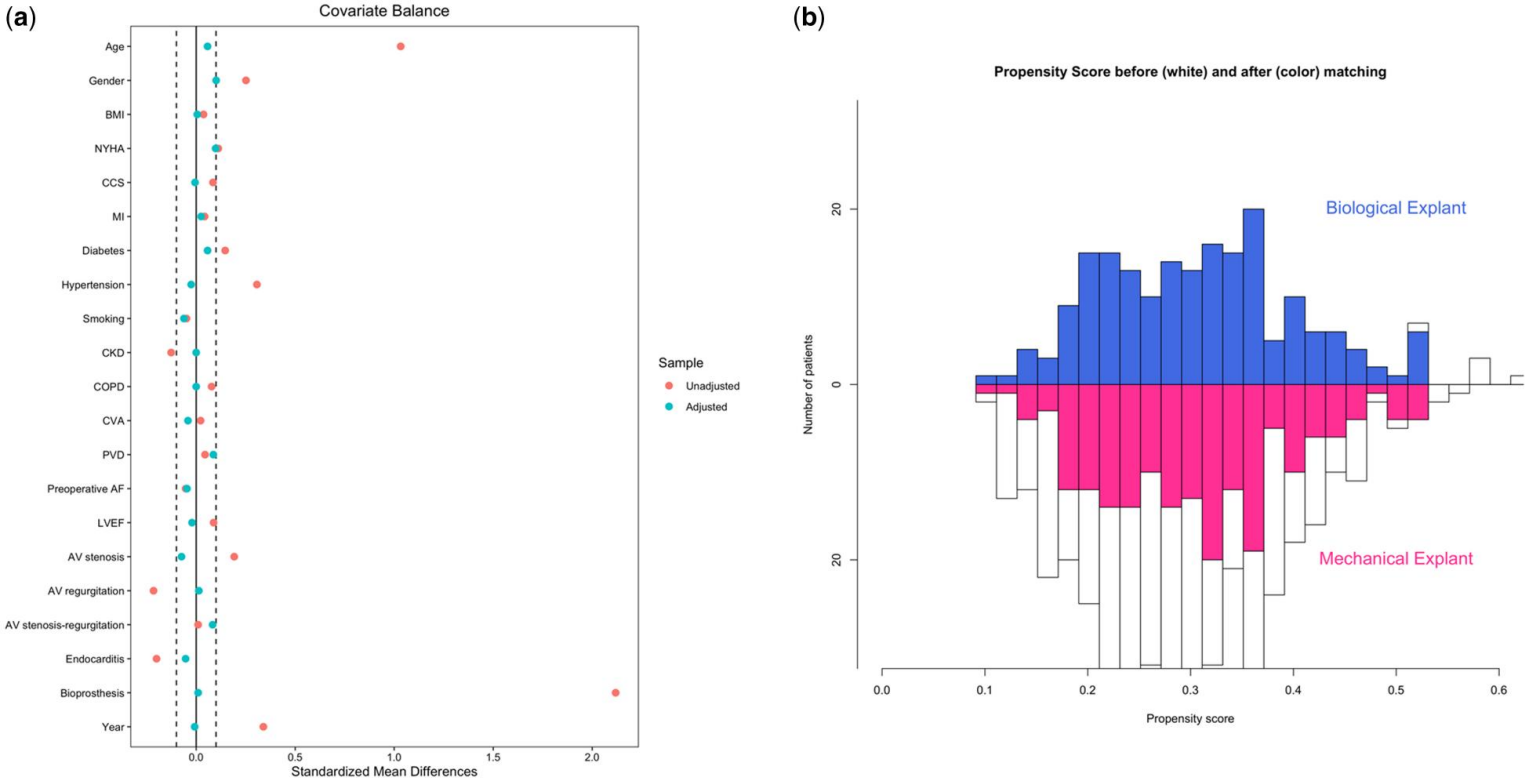
(**A**) Balance of propensity matching of patients undergoing Biological versus Mechanical Explant. (**B**) Propensity scores before (white) and after matching (coloured) to assess efficiency of propensity matching.

To assess the balance of covariates between the 2 groups after matching, standardized mean differences were computed. A value exceeding 0.10 was indicative of residual imbalance among variables. Furthermore, the quality of the match was scrutinized graphically through a Love plot of standardized mean differences (Fig. 2), which evaluated the balance of variables between the 2 groups. Additionally, a mirror plot illustrated the ‘common support area’, demonstrating the spectrum of propensity score values shared between the 2 groups (Fig. 2). Comparisons of continuous and categoric variables in the matched cohort were performed using the Wilcoxon signed rank test and the McNemar test, respectively, to account for paired membership of patients included in the sample.

In the matched cohort, we conducted comparisons of both continuous and categorical variables utilizing appropriate statistical tests, such as Pearson’s Chi-squared test, Fisher’s exact test and Kruskal–Wallis rank sum test, selecting the test that was most suitable for each specific type of variable. Logistic regression was conducted between in-hospital mortality and baseline as well as operative variables in the matched population to account for any residual imbalances that may exist after matching. The variables included were reoperative AVR, age, gender, body mass index, previous myocardial infarction, diabetes, Canadian Cardiovascular Society and NYHA scores, hypertension, history of smoking, chronic kidney disease, chronic obstructive pulmonary disease, prior cerebrovascular accident, peripheral vascular disease, preoperative atrial fibrillation, LVEF, endocarditis, type of ‘aortic’ valve implant, type of ‘aortic’ valve explant and the year of the operation. Effect estimates were presented as odds ratios (ORs) on a forest plot along with their corresponding 95% confidence intervals (CIs) and significance. The selection of independent variables from the list above for inclusion in the final model was guided by the Akaike Information Criterion, employing 500 bootstrap datasets [11]. Variables that exhibited statistical significance in at least 50% of the iterations were retained in the final model.

In all analyses, reoperative mechanical valve replacement served as the reference category. Statistical significance was determined at a threshold of *P*-value of <0.05 for all analyses. The statistical analysis was executed using R version 4.0.0.

## RESULTS

**Figure 3:**
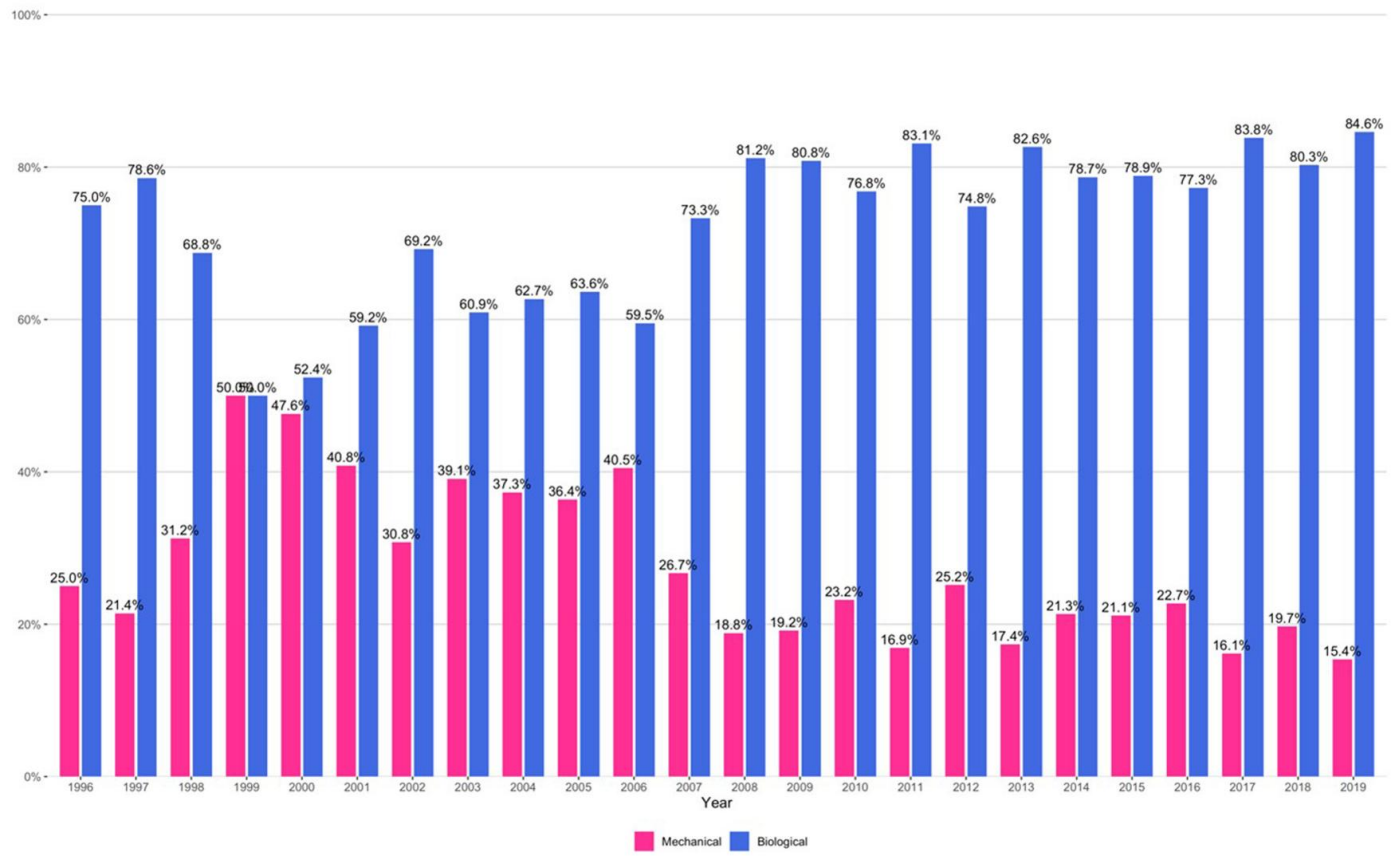
Cases trend for mechanical and biological aortic valve explants.

During the study period, 2371 reoperative AVRs were carried out in the UK where the previous operation was either a mechanical or a bioprosthetic valve. Of these, 592 (25%) were mechanical valves and 1779 (75%) were bioprosthetic valves. The number of patients with a mechanical valve presenting for reoperation was 25% in the year 1996 which decreased to 15.4% in 2019. The number of bioprosthetic valves which were presented for reoperation constituted the majority of the reoperations throughout the study. In 1996, while 75% of the reoperations were on a bioprosthetic valve, this further increased to 84.6% by 2019 (Fig. 3). However, in-hospital mortality decreased during the study period in both biological and mechanical groups from their inception (Supplementary Material, Fig. S2).

There were 880 (37.1%) women in the study population. A history of previous cerebrovascular accident was elicited in 228 (9.6%) cases and preoperative atrial fibrillation was present in 309 (13.0%) patients. In the study population, 271 (11.4%) presented with NYHA Class IV symptoms, 57 (2.2%) presented with cardiogenic shock and 49 (2.06%) needed preoperative ventilation. LVEF was ≤30% in 661 (27.8%) patients, A detailed comparison of preoperative variables among different valve types is provided in Table 1.

**Table 1:** Unmatched Comparison of Baseline Characteristics

Baseline characteristics	Mechanical	Bio-tissue	*P*-value
Number	592	1779	
Age (years), median (IQR)	60.55 (52.20, 67.32)	72.30 (65.65, 77.55)	<0.001
Female, *n* (%)	165 (27.9)	715 (40.2)	<0.001
NYHA functional class, *n* (%)			<0.001
1	99 (16.7)	222 (12.5)	
2	204 (34.5)	582 (32.7)	
3	218 (36.8)	775 (43.6)	
4	71 (12.0)	200 (11.2)	
CCS grade, *n* (%)			<0.001
0	366 (61.8)	1031 (58.0)	
1	96 (16.2)	301 (16.9)	
2	92 (15.5)	315 (17.7)	
3	32 (5.4)	100 (5.6)	
4	6 (1.0)	32 (1.8)	
EuroSCORE II, median (IQR)	3.43 (2.50, 6.36)	4.61 (3.12, 7.74)	<0.001
Body mass index (kg/m^2^), median (IQR)	27.43 (23.81, 30.36)	27.53 (24.50, 30.41)	<0.001
Years between operations, median (IQR)	6.80 (1.88, 13.98)	6.86 (2.39, 11.38)	<0.001
Diabetes mellitus, *n* (%)			<0.001
No	533 (90.0)	1515 (85.2)	
On diet control	15 (2.5)	51 (2.9)	
Oral medication	36 (6.1)	160 (9.0)	
Insulin	8 (1.4)	53 (3.0)	
Hypertension, *n* (%)	274 (46.3)	1089 (61.2)	<0.001
Smoking, *n* (%)			<0.001
Never smoked	276 (46.6)	800 (45.0)	
Former smoker	254 (42.9)	874 (49.1)	
Current smoker	62 (10.5)	105 (5.9)	
Chronic kidney disease, *n* (%)	37 (6.2)	68 (3.8)	<0.001
Chronic obstructive pulmonary disease, *n* (%)	62 (10.5)	233 (13.1)	0.015
Previous myocardial infarction, *n* (%)	33 (5.6)	118 (6.6)	0.135
Previous cerebrovascular attack, *n* (%)	54 (9.1)	174 (9.8)	0.019
Peripheral vascular disease, *n* (%)	33 (5.6)	119 (6.7)	0.084
Preoperative atrial fibrillation, *n* (%)	85 (14.4)	224 (12.6)	0.001
Left ventricular ejection fraction, *n* (%)			<0.001
Poor <30%	185 (31.2)	476 (26.8)	
Moderate 30–50%	6 (1.0)	40 (2.2)	
Good ≥50%	401 (67.7)	1263 (71.0)	
Cardiogenic shock, *n* (%)	18 (3.0)	38 (2.1)	<0.001
Preoperative inotropes, *n* (%)	31 (5.2)	72 (4.0)	<0.001
Preoperative ventilation, *n* (%)	16 (2.7)	33 (1.9)	<0.001

After propensity matching, the 2 groups where the mechanical and bioprosthetic valves were explanted included 324 patients each. The groups were well matched for all preoperative variables including EuroSCORE II, duration between the index and the reoperation, as well as presence of neurological, respiratory, cardiac and other comorbidities (Table 2). During the reoperation, a bioprosthetic valve was implanted in 135 (41.7%) cases out of 324 where a mechanical valve was explanted. In patients where a bioprosthetic valve was explanted, a similar number of patients, 136 (42%), received another bioprosthetic valve. The incidence of endocarditis was 29.6% (*n* = 96) in the mechanical valve explant group and 27.5% (*n* = 89) in the bioprosthetic group, *P* = 0.602. The only significant difference between the groups was a longer cross-clamp time in the mechanical valve explant group which was longer by a median of 5 min (Table 3).

**Table 2:** Propensity-matched comparison of baseline characteristics

Baseline characteristics	Mechanical	Bio-tissue	*P*-value	Unmatched SMD	Matched SMD
Number	324	324			
Age (years), median (IQR)	62.90 (55.50, 69.35)	65.20 (54.18, 72.90)	0.300	0.97	0.0573
Female, *n* (%)	84 (25.9)	100 (30.9)	0.185	0.262	0.1007
NYHA functional class, *n* (%)			0.702	0.159	0.098
1	58 (17.9)	48 (14.8)			
2	92 (28.4)	90 (27.8)			
3	129 (39.8)	136 (42.0)			
4	45 (13.9)	50 (15.4)			
CCS grade, *n* (%)			0.561	0.101	−0.0059
0	201 (62.0)	193 (59.6)			
1	47 (14.5)	61 (18.8)			
2	48 (14.8)	47 (14.5)			
3	23 (7.1)	17 (5.2)			
4	5 (1.5)	6 (1.9)			
EuroSCORE II, median (IQR)	3.79 (2.58, 6.87)	4.12 (2.76, 6.91)	0.124	0.161	0.106
Body mass index (kg/m^2^), median (IQR)	27.14 (23.63, 30.41)	27.58 (24.36, 29.84)	0.914	0.036	0.0054
Years between operations, median (IQR)	7.12 (2.48, 14.20)	7.62 (3.24, 12.71)	0.731	0.204	0.121
Diabetes mellitus, *n* (%)			0.522	0.164	0.0573
No	291 (89.8)	284 (87.7)			
On diet control	5 (1.5)	9 (2.8)			
Oral medication	22 (6.8)	21 (6.5)			
Insulin	6 (1.9)	10 (3.1)			
Hypertension, *n* (%)	160 (49.4)	156 (48.1)	0.798	0.303	−0.0253
Smoking, *n* (%)			0.112	0.187	−0.062
Never smoked, *n* (%)	148 (45.7)	146 (45.1)			
Former smoker, *n* (%)	141 (43.5)	157 (48.5)			
Current smoker, *n* (%)	35 (10.8)	21 (6.5)			
Chronic kidney disease, *n* (%)	23 (7.1)	23 (7.1)	1.000	0.111	0
Chronic obstructive pulmonary disease, *n* (%)	36 (11.1)	36 (11.1)	1.000	0.081	0
Previous myocardial infarction, *n* (%)	18 (5.6)	20 (6.2)	0.868	0.044	0.0248
Previous cerebrovascular attack, *n* (%)	33 (10.2)	29 (9.0)	0.677	0.023	−0.0416
Peripheral vascular disease, *n* (%)	14 (4.3)	21 (6.5)	0.281	0.046	0.0865
Preoperative atrial fibrillation, *n* (%)	49 (15.1)	44 (13.6)	0.657	0.052	−0.0465
Preoperative ventricular arrythmias, *n* (%)	1 (0.3)	0 (0.0)	1.000	0.048	0.079
Left ventricular ejection fraction, *n* (%)			0.435	0.134	−0.0209
Moderate–Good ≥30%	220 (67.9)	219 (67.6)			
Poor <30%	104 (32.1)	105 (32.4)			
Cardiogenic Shock, *n* (%)	7 (2.2)	10 (3.1)	0.628	0.057	0.058
Preoperative inotropes, *n* (%)	13 (4.0)	21 (6.5)	0.186	0.057	0.111
Preoperative ventilation	9 (2.8)	10 (3.1)	1	0.057	0.018

**Table 3:** Operative characteristics

	Mechanical^a^	Bio-tissue^a^	*P*-value^b^	Missing (%)
Number	324	324		
Median sternotomy	323 (99.7)	324 (100.0)	1	0
Partial sternotomy	1 (0.3)	0 (0.0)	1	0
Aortic valve implant			1	0
Bioprosthesis	135 (41.7)	136 (42.0)		
Mechanical valve	189 (58.3)	188 (58)		
Aortic valve size	23.00 (21.00, 24.00)	23.00 (21.00, 23.00)	0.2	17.9
Cardiopulmonary bypass time	106.00 (83.25, 150.00)	107.50 (84.75, 144.00)	0.539	4.6
Cross-clamp time	79.00 (63.00, 106.00)	74.00 (60.00, 98.00)	0.035	4.9
Endocarditis	96 (29.6)	89 (27.5)	0.602	0

a *n* (%); median (IQR).

b Pearson’s Chi-squared test; Fisher’s exact test; Kruskal–Wallis rank sum test.

There was no difference in mortality between patients where the explanted valve was mechanical or a bioprosthetic valve following reoperative AVR [23 (7.1%) vs 19 (5.9%), *P* = 0.632]. No differences were observed among the 2 groups in terms of re-exploration for bleeding (*P* = 0.13), postoperative neurological events (*P* = 0.26) or a need for postoperative dialysis (*P* = 0.2; Table 4). Mortality was higher in the presence of endocarditis with both mechanical [11 (11.4%) vs 12 (5.2%)] and bioprosthetic valves [10 (11.2%) vs 9 (3.8%)]. Patients with severe or very severe impairment of left ventricular function ‘above the age of 70’ were operated with a mortality of 10.8% (38/351) compared to the mortality observed in the entire unmatched population of 5.1% (123/2371) or 6.4% (42/648) of the matched cohort. The highest mortality for the entire cohort was 18.8% in the year 1998. Towards the end of the study, it was 6.82% in the year 2018 and 0% in the first 3 months of 2019.

**Table 4:** Propensity-matched comparison of postoperative outcomes

Postoperative outcomes	Mechanical^a^	Bio-tissue^a^	*P*-value^b^
Number	324	324	
Length of stay (days)	9.00 (6.00, 15.00)	9.00 (6.00, 16.00)	0.835
Return to theatre for bleeding	29 (9.0)	18 (5.6)	0.13
Cerebrovascular accident			0.269
None	317 (97.8)	312 (96.3)	
Transient ischaemic attack	2 (0.6)	1 (0.3)	
Stroke	5 (1.5)	11 (3.4)	
Deep sternal wound infection	1 (0.3)	0 (0.0)	1
Dialysis	20 (6.2)	12 (3.7)	0.204
Postoperative IABP use	2 (2.0)	1 (0.8)	0.832
Mortality	23 (7.1)	19 (5.9)	0.632

a *n* (%); median (IQR).

b Pearson’s Chi-squared test; Fisher’s exact test; Kruskal–Wallis rank sum test.

Multivariable logistic regression carried out to identify independent risk factors for in-hospital mortality in the unmatched groups confirmed that the type of valve implanted at the primary operation was not a risk factor for mortality (OR 0.62, 95% CI 0.37–1.05; *P* = 0.1). Age (OR 1.03, 95% CI 1.01–1.05; *P* < 0.05), left ventricular ejection fraction (OR 1.62, 95% CI 1.08–2.42; *P* < 0.05), creatinine ≥ 200 mg/dl (OR 2.21, 95% CI 1.17–4.04; *P* < 0.05) and endocarditis (OR 2.66, 95% CI 1.71–4.14; *P* < 0.05) were independent risk factors for mortality in reoperative aortic valve surgery. Similarly, mortality was found to decrease significantly across the years (OR 0.95, 95% CI 0.91–0.99; *P* < 0.05). Multivariable Logistic Regression Parsimonious set of predictors for Mortality based on Akaike information criterion showed that in 83.4% of the bootstrap iterations, the left ventricular ejection fraction was found to be significant compared to age (72.6%), and endocarditis (71.4%) of iteration (Table 5).

**Table 5:** Multivariable logistic regression parsimonious set of predictors for mortality based on Akaike information criterion using 500 bootstrap datasets at alpha = 0.05

Risk factor	Statistically significant (%)
Left ventricular ejection fraction	83.4
Age	72.6
Endocarditis	71.4
Year	61.6
Preoperative atrial fibrillation	52.6

Statistically significant: percentage of times risk factor was found significant.

## DISCUSSION

In our study, no significant differences were found in mortality or postoperative morbidity between patients undergoing reoperation on mechanical or bioprosthetic valves. The type of valve used during the initial operation was not a significant determinant of mortality. Instead, age, LVEF, renal impairment (creatinine ≥ 200 mg/dl) and the presence of endocarditis emerged as independent risk factors for mortality in reoperative AVR. Bioprosthetic valves were more frequently explanted in cases requiring reoperation throughout the study.

Although the overall volume of reoperative aortic valve surgeries has witnessed a slight decline in the UK [12], our study reveals a notable increase in the proportion of patients necessitating reoperation due to bioprosthetic valve, accounting for 84.6% of all isolated primary aortic valve reoperations conducted in the UK by the end of our study period. This trend may, therefore, reflect the global increase in the utilization of bioprosthetic valves for initial AVRs, as observed in prior studies [13, 14].

Nevertheless, it is widely acknowledged that the risk of undergoing reoperation is higher for bioprosthetic valves due to structural valve degeneration, whereas mechanical valves are conventionally recognized for their greater durability [15]. Our study revealed a paradoxical observation that among patients necessitating reoperation, the mean duration between the primary and subsequent procedures appeared to be comparable in both the matched and unmatched comparison of mechanical and bioprosthetic valves. Contemporary series have reported the temporal interval between the primary aortic valve operation and subsequent reoperation, as a median duration of 6.2 years [16]. In our series, we observed a median duration of 7.12 years for mechanical valves and 7.62 years for bioprosthetic valves.

Our study also introduces a novel perspective that contradicts the prevailing literature in this field. Notably, our findings indicate that mortality rates during reoperation remained similar, regardless of whether the initially implanted valve was mechanical or bioprosthetic. In contrast, a majority of prior studies have consistently reported higher mortality rates in reoperations involving mechanical valves [1, 3, 4, 6], with some studies even revealing nearly a 3-fold increase in risk [3, 6]. Previous research has documented mortality rates ranging from 12.4% to 26.1% for reoperations on mechanical valves and 6.8% to 13.2% for those involving bioprosthetic valves [2–6]. In our study, in-hospital mortality was notably lower, at 7.1% for mechanical valves and 5.9% for bioprosthetic valves.

The commonest causes for reoperation in the mechanical valves included endocarditis, valve dehiscence, para-prosthetic leaks, valve thrombosis, pannus formation and intrinsic valve failure. In bioprosthetic valves, structural valve degeneration was the commonest cause, followed by endocarditis and valve dehiscence. Several factors contribute to the notably reduced mortality observed in our study over an extended period, even in the face of variations in institutional caseloads and expertise. Firstly, the global risk associated with reoperation has steadily diminished, both on a worldwide scale and within the UK. Many of the earlier studies that reported higher mortality rates for mechanical valves were published between 1994 and 2007 [1–6]. In contrast, more recent series have documented considerably lower mortality rates, ranging from 2.4% to 9.1% [16–20]. The overall mortality in the UK for reoperative AVR has also been reported as 3.1% [12]. Thus, it is evident that the risk of reoperation has progressively declined in tandem with the advancement of medical knowledge and the accumulation of experience and expertise in the field. Second, it is noteworthy that several of these studies did not differentiate between reoperative isolated AVR and cases involving concomitant procedures. Consequently, the comparison of outcomes between mechanical and bioprosthetic valves is frequently confounded by the presence of concomitant procedures, such as coronary artery bypass grafting [1, 3], aortic surgery [3, 17] or multiple valve replacements [1, 2, 4]. In our study, we deliberately excluded patients requiring concomitant surgery at the time of reoperation from the analysis, resulting in a homogeneous cohort of patients necessitating isolated aortic valve reoperations. This approach allowed for a more precise evaluation of the outcomes associated with different valve types. Despite heterogeneity, there are few studies which also corroborate our observations on the lack of difference in mortality influenced by the explanted valve type.

Our study provided important information on the risk factors for in-hospital mortality in this specific cohort of patients undergoing isolated reoperative aortic valve surgery where the previous operation was an AVR with a biological or mechanical valve. Endocarditis, creatinine ≥ 200 mg/dl, left ventricular ejection fraction and age emerged as the most important risk factor.

Endocarditis was identified as the primary indication for reoperation in 25.9% of our study participants, aligning with findings from prior research [1, 3, 8, 16, 19, 21]. Notably, in one of these studies, endocarditis accounted for a substantial 38% of all reoperations [16]. Thus, although there is some suggestion that the occurrence of endocarditis as a triggering factor for reoperations may be somewhat reduced in cases involving bioprosthetic valves [1, 22], it is crucial to recognize the significance of endocarditis as a primary driver for reoperations, irrespective of the type of valve originally implanted. A creatinine >200 mg/dl in our study was a risk factor for mortality in our study. This is consistent with previous reports which have reported that chronic renal impairment leads to an increase in both morbidity and early as well as late mortality in patients undergoing reoperative AVR [12, 16].

The role of LVEF as a risk factor for early mortality has been widely documented in numerous studies [19, 21, 23, 24]. However, one recent study did not find LVEF to be a significant risk factor [16]. It is important to consider that in this particular study, nearly one-fourth of patients undergoing reoperation also had concurrent procedures performed, and a substantial portion of patients during the initial operation underwent multiple other procedures [16]. In contrast, our study revealed that a lower LVEF was associated with a mortality rate exceeding 3 times that of patients with higher LVEF. In 2 previous studies, the mortality rate was reported to be 9 times higher in the context of reoperative surgery; however, it is vital to recognize that both of these studies included a significant proportion of patients who underwent concomitant procedures [19, 21]. Therefore, it is likely that our findings provide a more accurate representation of the role of LVEF in determining mortality within this patient cohort.

Age consistently emerges as a reported risk factor for mortality in the context of reoperative AVR, corroborated by previous research [3, 16, 25], Nevertheless, it is worth noting that excellent outcomes have been documented in elderly patients who have undergone reoperative surgery [26]. While age alone should not be the exclusive factor in deciding against reoperation, our findings emphasize that reoperative surgery in elderly patients with LVEF impairment carries an exceptionally high risk of mortality. Consequently, alternative approaches such as valve-in-valve transcatheter AVR, where feasible, may be considered in the elderly patients with multiple risk factors.

### Limitations

The present study conducted a retrospective analysis of data extracted from the NICOR database, which serves as an invaluable resource for investigating nationwide cardiac surgical outcomes. Nevertheless, certain limitations inherent to retrospective data analysis deserve acknowledgment. In addressing missing data, the study employed imputation techniques for both categorical and continuous variables. While these methods aim to mitigate the impact of missing data, they may introduce a measure of imprecision into the analytical process. To enhance the comparability of the study cohorts, propensity score matching was meticulously applied, achieving commendable balance between the groups. However, the influence of unmeasured confounders, if present, remains beyond the scope of the analysis. In the pursuit of a refined and homogenous comparison, several potential confounders were deliberately excluded. Although this approach affords an exceptionally accurate comparison within the specified study groups, it simultaneously limits the broader generalizability of the findings. It is essential to emphasize that the study findings are not extrapolatable to patient populations undergoing concomitant surgical procedures in conjunction with AVR or those in whom the primary operation encompassed interventions other than AVR with mechanical or bioprosthetic valves. Furthermore, the study primarily centres on in-hospital outcomes, thus precluding the assessment of longer-term follow-up data encompassing late mortality, valve-related complications and patient-reported quality-of-life measures. Even though our study aimed at assessing the impact of explanted valve type on early mortality reporting, our future work will investigate the linkage of this national dataset with Hospital Episode Statistics dataset to investigate effects on long-term outcomes.

Notwithstanding these acknowledged limitations, this study makes a substantive and noteworthy contribution by providing valuable insights into the outcomes of reoperative aortic valve surgery, with a particular focus on the influence of the initially implanted valve type. Significantly, it underscores the pivotal roles of age, LVEF and the presence of endocarditis as robust and independent risk factors for mortality within this distinctive patient population. These insights hold considerable promise for informing clinical decision-making and enhancing patient care in the realm of reoperative aortic valve surgery.

## CONCLUSIONS

In summary, our study focused on isolated first-time reoperative AVRs, revealing that the type of valve initially implanted, be it mechanical or biological, had no substantial impact on mortality rates. Our approach of excluding patients requiring concurrent procedures allowed for a more precise evaluation of outcomes related to different valve types. Our analysis affirmed age, LVEF, creatinine >200 and the presence of endocarditis as the key independent risk factors for mortality. Our study also reports an increase in the proportion of patients requiring reoperation due to bioprosthetic valve issues. This trend may reflect the global rise in the use of bioprosthetic valves for initial AVRs. While age alone should not preclude reoperation, our findings underscore the significantly elevated risk of mortality in elderly patients with impairment of the left ventricular function.

## Supplementary Material

ezae031_Supplementary_Data

## Data Availability

The data underlying this article were provided by National Institute of Cardiovascular Outcomes Research (NICOR). Data can be shared on request to the corresponding author subject to permission from NICOR.

## Notes

Presented at the 37th Annual Meeting of the European Association for Cardio-Thoracic Surgery, Vienna, Austria, 6 October 2023.

## ABBREVIATIONS

AVR: Aortic valve replacement
CI: Confidence interval
HCRW: Health and Care Research Wales
LVEF: Left ventricular ejection fraction
NICOR: National Institute of Cardiovascular Outcomes Research
NYHA: New York Heart Association
OR: Odds ratio
