# Corrigendum to: Impact of explanted valve type on aortic valve reoperations: nationwide UK experience

**DOI:** 10.1093/ejcts/ezae340

**Published:** 2024-09-27

This is a corrigendum to: Pradeep Narayan, Tim Dong, Arnaldo Dimagli, Daniel P Fudulu, Jeremy Chan, Shubhra Sinha, Gianni D Angelini, Impact of explanted valve type on aortic valve reoperations: nationwide UK experience, *European Journal of Cardio-Thoracic Surgery*, Volume 65, Issue 2, February 2024, https://doi.org/10.1093/ejcts/ezae031

The following changes have been made to the originally published manuscript. A statement of funding has been added to end of the text in the section **Funding**: “BHF Turing SP/19/7/34810”.

