# Supplementary material for: Impact of explanted valve type on aortic valve reoperations: nationwide UK experience
Source: Eur J Cardiothorac Surg. 2024 Feb 1;65(2):ezae031. doi: 10.1093/ejcts/ezae031 (PMC10902681; doi:10.1093/ejcts/ezae031)
Supplement: ezae031_Supplementary_Data [file ezae031_supplementary_data.docx]

Figure S1. Trend in mortality across groups from 1996-2019. Note, data is up to March 2019.


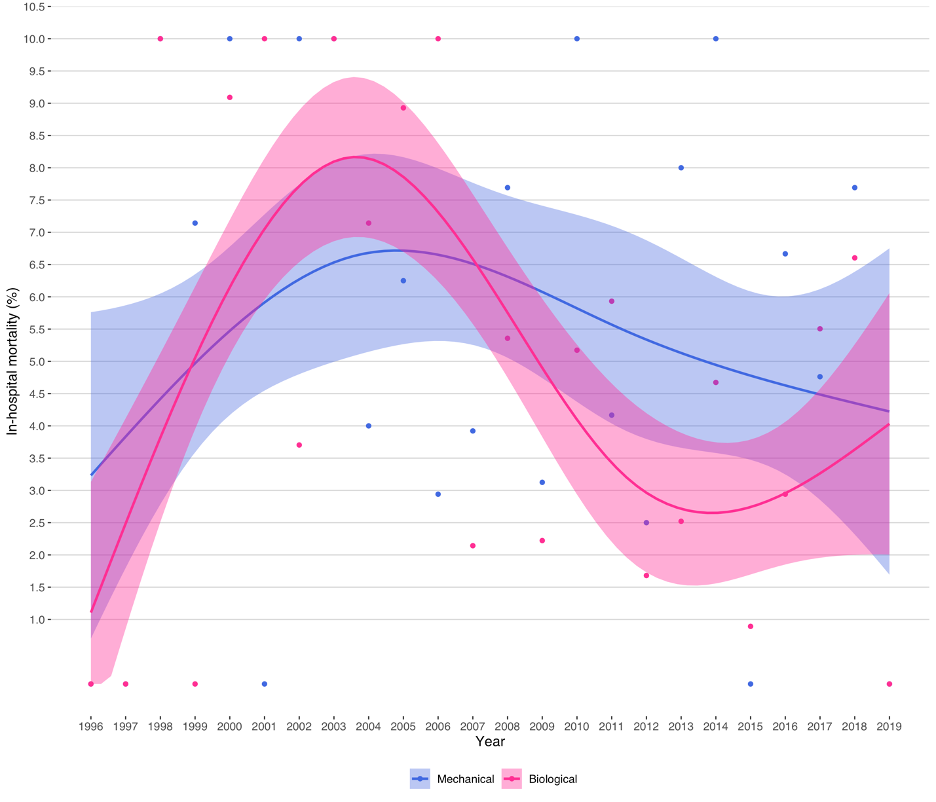


Figure S2. Comparison of propensity scores against inverse probability of treatment weighting (IPTW) distributions.


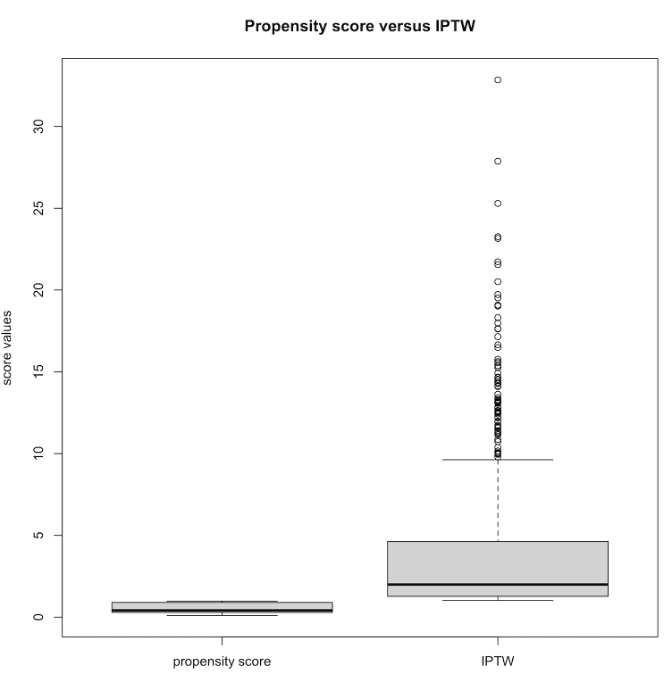


| **Supplementary Table S1: Unmatched comparison of Operative characteristics of Mechanical and Bioprosthetic valves** | | | | | | |
| --- | --- | --- | --- | --- | --- | --- |
|  | **Mechanical** | **Bio-tissue** | **P Value** | **test** | **SMD** | **Missing (%)** |
| Number | 592 | 1779 |  |  |  |  |
| Median Sternotomy | 591 ( 99.8) | 1772 ( 99.6) | 0.684 |  | 0.042 | 0 |
| Partial Sternotomy | 1 ( 0.2) | 7 ( 0.4) | 0.684 |  | 0.042 | 0 |
| Aortic valve implant |  |  | <0.001 |  | 1.77 | 0 |
| Bioprosthesis | 139 ( 23.5) | 1587 ( 89.2) |  |  |  |  |
| Mechanical valve | 453 (76.5) | 192 (10.8) |  |  |  |  |
| Aortic valve size | 23.00 [21.00, 25.00] | 23.00 [21.00, 23.00] | 0.029 | nonnorm | 0.114 | 15.1 |
| Cardio-Pulmonary Bypass Time | 105.00 [84.50, 145.00] | 98.00 [78.00, 125.00] | <0.001 | nonnorm | 0.241 | 4.2 |
| Cross Clamp Time | 78.50 [63.00, 105.00] | 71.00 [57.00, 90.00] | <0.001 | nonnorm | 0.279 | 4.3 |
| Endocarditis | 169 ( 28.5) | 364 ( 20.5) | <0.001 |  | 0.189 | 0 |
| *^1^*n (%); Median (IQR)  *^2^*Pearson's Chi-squared test; Fisher's exact test; Kruskal-Wallis rank sum test | | | | | | |

| **Supplementary Table S2: Unmatched comparison of postoperative outcomes of Mechanical and Bioprosthetic valves** | | | |
| --- | --- | --- | --- |
|  | **Mechanical** | **Bio-tissue** | **P Value***^2^* |
| Number | 592^1^ | 1779^1^ |  |
| Length of stay-days | 9.00 [6.00, 15.00] | 9.00 [6.00, 14.00] | 0.217 |
| Return to theatre for bleeding | 40 ( 6.8) | 129 ( 7.3) | 0.754 |
| Cerebrovascular accident |  |  | 0.309 |
| None | 579 (97.8) | 1735 (97.5) |  |
| Transient ischemic attack | 5 ( 0.8) | 8 ( 0.4) |  |
| Stroke | 8 ( 1.4) | 36 ( 2.0) |  |
| Deep sternal wound infection | 2 ( 0.3) | 1 ( 0.1) | 0.316 |
| Dialysis | 28 ( 4.7) | 84 ( 4.7) | 1 |
| Post-op IABP use | 3 ( 1.7) | 17 ( 2.2) | 0.852 |
| Mortality | 37 ( 6.2) | 86 ( 4.8) | 0.216 |
|  |  |  |  |
| *^1^*n (%); Median (IQR)  *^2^*Pearson's Chi-squared test; Fisher's exact test; Kruskal-Wallis rank sum test | | | |
